# Silencing of a large microRNA cluster on human chromosome 14q32 in melanoma: biological effects of mir-376a and mir-376c on insulin growth factor 1 receptor

**DOI:** 10.1186/1476-4598-11-44

**Published:** 2012-07-02

**Authors:** Liron Zehavi, Roi Avraham, Aviv Barzilai, Dalia Bar-Ilan, Roy Navon, Yechezkel Sidi, Dror Avni, Raya Leibowitz-Amit

**Affiliations:** 1 Laboratory of Molecular Cell Biology, Cancer Research Center and Department of Medicine C, Sheba Medical Center, Tel Hashomer, Israel; 2 Sackler School of Medicine, Tel Aviv University, Tel Aviv, Israel; 3 Department of Biological Regulation, Weizmann Institute of Science, Rehovot, Israel; 4 Institute of Pathology, Sheba Medical Center, Tel Hashomer, Israel; 5 Department of Dermatology, Sheba Medical Center, Tel Hashomer, Israel; 6 Agilent Laboratories, Tel Aviv, Israel; 7 Institute of Oncology, Sheba Medical Center, Tel Hashomer, 52621, Israel

**Keywords:** microRNA, Melanoma, IGF1R, mir-376a, mir-376c, Epigenetics

## Abstract

**Background:**

Metastatic melanoma is a devastating disease with limited therapeutic options. MicroRNAs (miRNAs) are small non coding RNA molecules with important roles in post-transcriptional gene expression regulation, whose aberrant expression has been implicated in cancer.

**Results:**

We show that the expression of miRNAs from a large cluster on human chromosome 14q32 is significantly down-regulated in melanoma cell lines, benign nevi and melanoma samples relative to normal melanocytes. This miRNA cluster resides within a parentally imprinted chromosomal region known to be important in development and differentiation. In some melanoma cell lines, a chromosomal deletion or loss-of-heterozygosity was observed in the cis-acting regulatory region of this cluster. In several cell lines we were able to re-express two maternally-induced genes and several miRNAs from the cluster with a combination of de-methylating agents and histone de-acetylase inhibitors, suggesting that epigenetic modifications take part in their silencing. Stable over-expression of mir-376a and mir-376c, two miRNAs from this cluster that could be re-expressed following epigenetic manipulation, led to modest growth retardation and to a significant decrease in migration in-vitro. Bioinformatic analysis predicted that both miRNAs could potentially target the 3'UTR of IGF1R. Indeed, stable expression of mir-376a and mir-376c in melanoma cells led to a decrease in IGF1R mRNA and protein, and a luciferase reporter assay indicated that the 3'UTR of IGF1R is a target of both mir-376a and mir-376c.

**Conclusions:**

Our work is the first to show that the large miRNA cluster on chromosome 14q32 is silenced in melanoma. Our results suggest that down-regulation of mir-376a and mir-376c may contribute to IGF1R over-expression and to aberrant negative regulation of this signaling pathway in melanoma, thus promoting tumorigenesis and metastasis.

## Background

Malignant melanoma is a devastating disease with a constantly increasing incidence worldwide and limited treatment options [1]. MicroRNAs (miRNAs) are small non coding RNA molecules that are generated within cells and play a role in post-transcriptional gene regulation [2]. It is becoming clear that aberrant expression of miRNAs has a role in cancerous transformation and progression [3]. Several miRNA-profiling studies in melanoma were published until now [4-6], but the picture emerging from these works is far from being clear.

A large miRNA cluster was recently shown to be down-regulated in ovarian cancer, and eight miRNAs in this cluster were identified as potential tumor suppressor genes [7]. Lately, this cluster was also implicated in gastro-intestinal stromal tumors (GISTs) [8] and in gliomas [9]. Additionally, mir-127 from this cluster was shown to have tumor suppressor function in a bladder cancer model [10]. This miRNA cluster lies within a parentally imprinted chromosomal area designated *Dlk1-Gtl2* in mouse or Dlk-Dio3 in human [11]. This area is of great developmental importance, exemplified by severe phenotypes associated with altered dosages of the genes within it in mice and humans [12]. The regulation of imprinting in this chromosomal locus is thought to be mediated, at least to some extent, by an intergenic differentially methylated region (IG-DMR) that is located centromeric to the imprinted region [13]. Indeed, this region was shown to be differentially methylated during embryonic development in humans [14]. Another regulatory region, located more telomeric, is designated 'MEG3-DMR'. Human studies performed on infants with uniparental dysomy of each of these DMRs imply that the IG-DMR and the MEG3-DMR function as imprinting control centers in the placenta and the body, respectively, with a hierarchical interaction for the methylation pattern in the body governed by the IG-DMR [15]. In mouse, deletion of IG-DMR from the maternally (but not the paternally) inherited chromosome causes bi-directional loss of imprinting of all genes in the cluster [11]. A meticulous characterization of all transcripts in this mouse locus demonstrated that the miRNAs within this cluster were exclusively expressed from the maternal chromosome. The other maternally expressed transcripts in this region (designated Meg3 and Meg8) were found to have exclusive patterns of expression, being detected only in brain, testis and skin [16]. Very recently, the expression of miRNAs from this region was found to be essential for maintaining full pluripotency of induced pluripotent stem cells [17].

Along the years, there have been few descriptions of chromosomal abnormalities in melanoma samples. 15 years ago, the translocation t(1;14)(q21;q32) was found in several of 20 melanoma samples taken from patients [18], and more than a decade later this chromosomal region was again found to be aberrant in some melanoma cell lines [19]. Recently, Zhang et al. determined DNA copy number abnormalities in 283 miRNA genes in three different cancer types (namely ovary, breast and melanoma) using comparative genomic hybridization, and showed loss of hetrozygocity (LOH) of the 14q32 miRNA cluster in 20% of the melanoma cell lines examined [20]. Nonetheless, this cluster has not been specifically implicated in melanoma so far.

We show here that this large miRNA cluster is silenced in melanoma cell lines, benign nevi and melanoma samples, and present data suggesting that both genetic and epigenetic mechanisms may take part in this silencing. We provide data showing that re-expression of mir-376a and mir-376c, two miRNAs from this cluster, lead to attenuation of melanoma proliferation and migration. These two miRNAs target IGF1R, a tyrosine kinase receptor implicated in melanoma tumorigenesis and metastasis.

## Results

**Figure 1:**
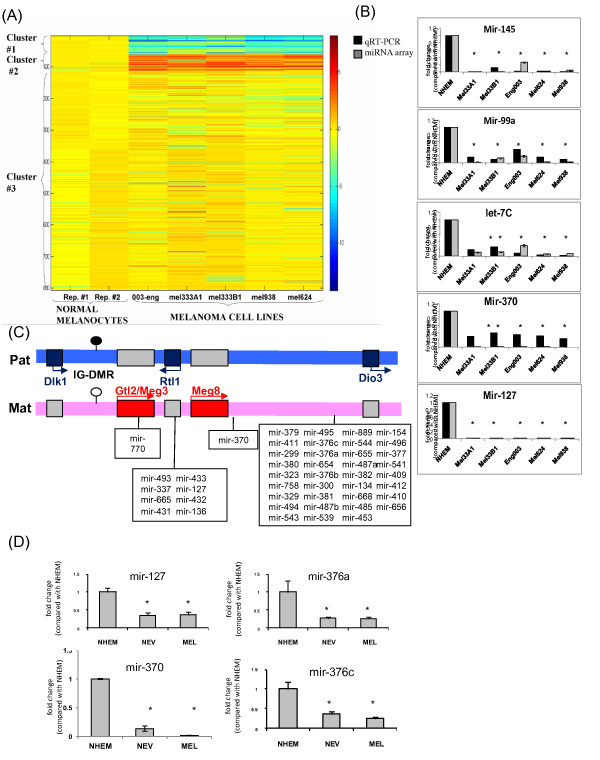
** The expression pattern of miRNAs in normal melanocytes and melanoma cell lines, and the organization of the 14q32 imprinted region.(A)** Heat map representing a cluster analysis of the logarithm of the normalized values of ~800 miRNAs embedded on a commercial array in normal melanocytes (two samples) and five different melanoma cell lines. **(B)** The expression levels of five different miRNAs using qRT-PCR (black) or data obtained from the array (grey). Data is represented as mean±SEM, * signifies p < 0.05 **(C)** The organization of the Dlk-Gtl2 region on human chromosome 14q32. The positions of miRNAs within this region are marked in rectangular boxes. The maternal allele is colored pink; the paternal allele is colored light-blue. The blue boxes signify paternally-expressed transcripts, and the red boxed signify maternally-expressed transcripts. The direction of transcription is marked with an arrow. The color of the hairpin at the IG-DMR illustrates the methylation status at the region; black – methylated, white hypo-methylated. **(D)** The expression levels of four miRNAs from the chromosome-14q32-cluster in normal melanocytes (NHEM), benign nevi (NEV) and melanoma samples (MEL) using qRT-PCR. Data is normalized relative to NHEM and is represented as mean±SEM, * signifies p < 0.05.

To compare the miRNA expression pattern between normal and malignant melanocytes, two samples of miRNAs produced from normal human epidermal melanocytes (NHEM) and miRNAs from five melanoma cell lines were hybridized to a commercial miRNAs array, using commercial placental miRNAs as positive control (Additional file 1). An unsupervised cluster anlysis of the logarithm of the normalized values using the k-means clustering algorithm showed that the two NHEM samples exhibit a very similar pattern of miRNAs expression, and that whereas the majority of miRNAs are not significantly altered between normal and malignant melanocytes (cluster #3, Figure 1A), there are two distinct groups of miRNAs that are either up-regulated or down-regulated in melanoma vs. melanocytes (cluster #2 and #1, respectively, Figure 1A). The expression pattern of several miRNAs from the array was validated by quantitative RT-PCR, and all were found to exhibit similar expression patterns as in the array (Figure 1B and results not shown).

Statistical analysis was undertaken to find miRNAs who exhibit the exact same pattern of expression (i.e. significant up-regulation or down-regulation) in *all* five melanoma cell lines compared to normal cells by using a student *t*-test with a p value < 0.0032 (corrected for multiple comparisons with a false discovery rate of 0.05). Using this very stringent criterion, only 58 miRNAs were found to be significantly altered between normal melanocytes and *all* five malignant melanoma cell lines, out of which 57 were significantly down-regulated in melanoma. Interestingly, of these 57 miRNAs, 27 were mapped to a large bipartite miRNA aggregate on chromosome 14. This cluster resides within a parentally imprinted region on chromosome 14q32 known to be imperative in development and differentiation (Figure 1C) [12,15]. We therefore decided to focus our present work on miRNAs from this large aggregate. Table 1 depicts the expression pattern of all miRNAs from this cluster.

**Table 1:** The expression levels of chromosome 14q32 miRNAs in melanoma

**miRNA that are not expressed in NHEM or in melanoma cell lines** (25)		**miRNA that are expressed in NHEM but not in melanoma cell lines** (**31**)		**miRNA that are expressed in NHEM and melanoma cell lines (6)**
Mir-127-5p	Mir-487a	Mir-127-3p	Mir-381	Mir-134
Mir-154*	Mir-493	Mir-136*	Mir-382	Mir-370
Mir-323-5p	Mir-496	Mir-136	Mir-409-3p	Mir-485-3p
Mir-323-3p	Mir-539	Mir-154	Mir-409-5p	Mir-494
Mir-369-5p	Mir-541	Mir-299-3p	Mir-410	Mir-654-5p
Mir-369-3p	Mir-541*	Mir-299-5p	Mir-411	Mir-770-5p
Mir-379*	Mir-544	Mir-329	Mir-411*	miRNAs with a discrepancy in expression between two NHEM samples (3)
Mir-380	Mir-655	Mir-337-3p	Mir-431	
Mir-380*	Mir-656	Mir-337-5p	Mir-432	
Mir-412	Mir-668	Mir-376a	Mir-487b	
Mir-432*	Mir-889	Mir376a*	Mir-493*	
Mir-433		Mir-376b	Mir-495	
Mir-453		Mir-376c	Mir-543	
Mir-485-5p		Mir-377	Mir-654-3p	Mir-300
		Mir-377*	Mir-758	Mir-431*
		Mir-379		Mir-665

The expression levels of 65 miRNAs from the large miRNA cluster on human chromosome 14q32.

We next compared the expression pattern of miRNAs from benign melanocytic nevi and melanoma samples taken from parrafin-embedded tissues to miRNAs from normal melanocytes (Additional file 2). In general, the expression patterns of miRNAs from benign nevi and malignant melanoma were very similar. Interestingly, chromosome 14q32 miRNAs were significantly over-represented in the cluster of miRNAs whose expression was significantly down-regulated in all melanoma and nevi. Whereas chromosome 14q32 miRNAs accounted for 7.6% of all miRNAs represented on the array (65 out of 851), they accounted for 23.5% of all the downregulated miRNAs (19 out of 81, p < 0.002 using chi-square, comparing the observed frequencies vs the expected). We validated our micro-array results by performing qRT-PCR on miRNA produced from two different samples of NHEM, fifteen samples of benign nevi and seven samples of melanoma. All miRNAs examined were significantly down-regulated in nevi and melanoma relative to NHEM (Figure 1D and results not shown).

Previous work in mice showed that silencing of the maternally-expressed genes (coding for mRNAs and miRNAs) could result from deletion of the regulatory IG-DMR region [11], whereas in an in-vitro human model system, epigenetic modifications led to re-expression of a miRNA from this cluster [10]. We thus hypothesized that the apparent miRNA silencing from chromosome 14 could be the result of a chromosomal deletion of the regulatory region, epigenetic modifications or a combination of the two. Since the IG-DMR is a control element for all imprinted genes on the maternal chromosome [11], and since the miRNAs are thought to be transcribed only from the maternal chromosome [16], we first designed a DNA copy number assay using quantitative real time PCR with two different probes taken from the IG-DMR region. As expected, there were two copies of each of the two probes in the DNA taken from a healthy human subject, in the DNA of normal melanocytes and in the DNA of most of the melanoma cell lines. However, there were two melanoma cell lines that exhibited only one copy of the IG-DMR DNA, and no copies of either of the two probes were detected in another cell line (Table 2). These results suggest that LOH or complete absence of the IG-DMR locus could explain the miRNA silencing in some, but not all, of the melanoma cell lines.

**Table 2:** IG-DMR copy number in melanoma cell lines

**Cell type**		**Calculated Copy number**
Normal DNA		2
Normal melanocytes (NHEM)		2
Melanoma Cell lines	Mel624	2
	14PA	2
	15AY	2
	mel33B1	2
	Mel28	1
	Eng003	1
	Mel33A1	0

The calculated IG-DMR copy number as assessed using qPCR in normal human DNA, DNA from normal melanocytes and seven melanoma cell lines.

**Figure 2:**
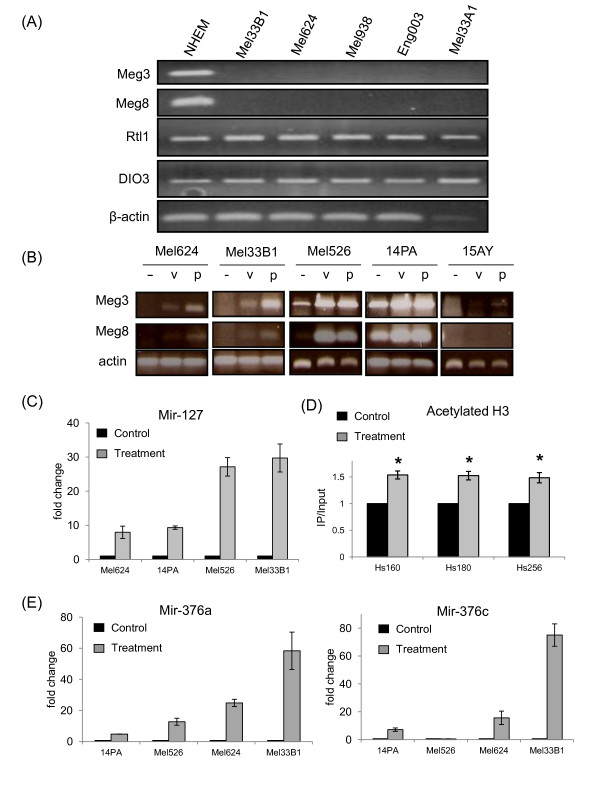
** Re-expression of maternally expressed transcripts from chromosome 14q32 following treatment with epigenetic modifiers and its effect on histone acetylation**. **(A)** The expression levels of the maternal (Meg3, Meg8) and paternal (Rtl1, Dio3) genes in normal melanocytes (NHEM) and melanoma cell lines. **(B)** The expression levels of Meg3 and Meg8 following treatment with 10 μM 5-AZA + 1.5 mM VPA (v) or 10 μM 5-AZA + 3 mM PBA (p) in five different cell lines. **(C)** The expression level of mir-127 in melanoma cells treated with epigenetic modifiers (10 μM 5-Aza and 3 mM PBA), as assessed by qRT-PCR and normalized relative to untreated cells. **(D)** ChIP assay of 14PA melanoma cells untreated or treated with 10 μM 5-Aza and 3 mM PBA. The normalized levels of chromosomal DNA at three different loci following immunoprecipitation with anti-acetylated histone 3 in control cells and in cells treated with epigenetic modifiers are shown. Data is represented as mean±SEM, * signifies p < 0.05. **(E)** The expression level of mir-376a and mir-376c in cells untreated or treated with 10 μM 5-Aza and 3 mM PBA.

We then set out to study the expression of genes from this locus. The maternally expressed genes Meg3 and Meg8, known to be selectively expressed only in brain, skin and testis [16], were detected in normal but not in malignant melanocytes. The paternally expressed genes Rtl1 and Dio3 were detected in all cell lines (Figure 2A). To assess whether epigenetic modifications take part in silencing from this cluster, we searched for conditions and combinations of epigenetic modifiers (de-methylating agents and HDAC inhibitors) that might bring about re-expression of the maternal genes from this cluster. Both maternal transcripts could be re-expressed after several days of treatment with a combination of the de-methylating agent 5 azacytidine (5-AZA) and the HDAC inhibitor valproic acid (VPA) but not with any of these agents alone (resuls not shown). The re-expression of the maternal expressed genes was observed in most of the cell lines examined, and was even more pronounced when using the HDAC inhibitor phenyl butyric acid (PBA; Figure 2B).

Re-expression of mir-127 was assessed using the same treatment conditions. Mir-127 could be induced between 8 to 30–fold using this treatment combination in all melanoma cell lines examined (Figure 2C). To verify that the treatment indeed led to epigenetic modifications in the vicinity of the regulatory region of the 14q32-cluster, chromatin immunoprecipitation (ChIP) using an anti-acetylated Histone 3 antibody was performed, showing that the addition of epigenetic modifiers increased the extent of histone acetylation in two different loci within the IG-DMR region and in another regulatory region located approximately 700 bp upstream of the mir-127 locus [10] (Figure 2D), suggesting that re-expression of these miRNAs is a result of a true epigenetic alteration in the cells.

We utilized the micro-array platform to see which other chromosome-14-miRNAs could be induced using the combination of HDAC inhibitors and de-methylating agents (Additional file 3). Interestingly, out of all 65 chromosome-14-miRNAs assessed in four melanoma cell lines, only five miRNAs were shown to be induced in any of the cell lines: mir-127-3p, mir-137, mir-376a (also designated mir-376a-3p), mir-376c and mir-485-3p. These five miRNAs, expressed in normal melanocytes, could not be further up regulated in these cells in response to epigenetic modifiers (Table 3). Four of these five miRNAs (mir-127-3p, mir-136, mir-376a and mir-376c) were found to be down-regulated but not entirely silenced in nevi and melanoma (Additional file 2). Results obtained with the more sensitive method of qRT-PCR verified that mir-376a, mir-376c and mir-136 can be significantly induced following treatment with epigenetic modifiers in most of the melanoma cell lines (Figure 2E and results not shown).

**Table 3:** Re-expression of chromosome 14q32 miRNAs following treatment with epigenetic modifiers

	Normal Human Melanocytes		Melanoma Cell lines							
			Mel33B1		14PA		Mel526		Mel624	
5aza + PBA	-	+	-	+	-	+	-	+	-	+
Mir-127	70.6	61.4	UD	UD	7.1	37.3	UD	9.5	UD	UD
Mir-136	52	44.4	UD	UD	7.9	30.8	UD	UD	UD	UD
Mir-376a	100.1	75.4	UD	UD	UD	12.4	UD	UD	UD	UD
Mir-376c	122.1	77.6	UD	UD	UD	10.4	UD	UD	UD	UD
Mir-485-3p	12.7	18.4	UD	23.9	37.7	38.6	31.1	19.2	UD	16

The expression levels of five chromosome-14q32 miRNAs in normal melanocytes and four melanoma cell lines as assessed by miRNA micro-array following treatment with epigenetic modifiers (10 μM 5-Aza and 3 mM PBA); *UD-undetected.* The miRNAs with significant differences between treatment and control are bolded

**Figure 3:**
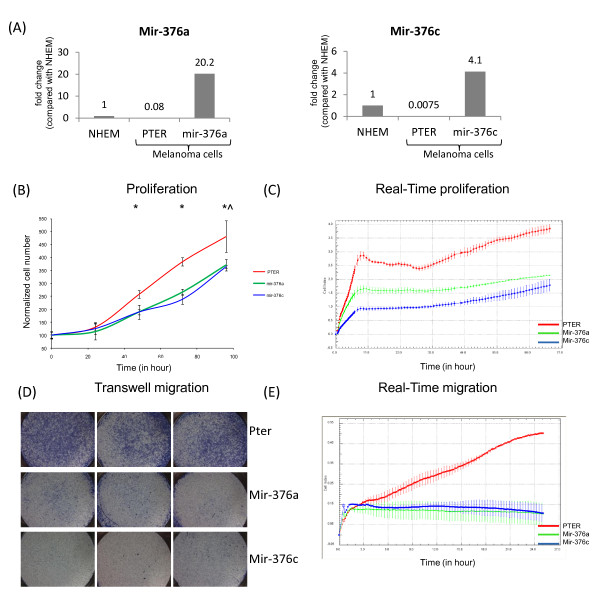
** The effect of stable expression of mir-376a and mir-376c on the proliferation and migration of melanoma cells.(A)** qRT-PCR analysis of mel33B1 melanoma cells after stable transfection with an empty pTER vector or vectors containing either mir-376a (left) or mir-376c (right). The relative expression levels are numerically depicted. **(B)** The growth of mel33B1 melanoma cell lines transfected with pTER, mir-376a and mir-376c was assessed using the crystal-violet method for 96 h. * signifies p < 0.05, *^ signifies p < 0.08 **(C)** The growth of mel33B1 melanoma cell lines transfected with pTER, mir-376a and mir-376c was assessed using the Xcellience ^TM^ real-time system. **(D)** The migration of these three cell lines was assessed using the in-vitro transwell method. Representative micrographs of the transwell membrane are seen. This experiment was repeated three times. **(E)** Cellular migration was assessed using the Xcellience ^TM^ real-time system within a 24 h period.

Mir-127 was previously shown to target BCL-6 in a bladder cancer model [10], so we first generated melanoma cell lines that ectopically express mir-127 in a stable manner. In our experimental system, mir-127 over-expression did not lead to a significant decrease in BCL-6 levels in melanoma cell lines, nor did it lead to a significant change in melanoma cell line proliferation or migration in vitro (results not shown). We therefore decided to focus on other miRNAs whose expression was shown to be down-regulated but not entirely absent in melanoma and as a first step generated melanoma cell lines that ectopically express either mir-376a or mir-376c (Figure 3A).

Cells over-expressing either mir-376a or mir-376c exhibited attenuated growth relative to pTER-transfected control cells (Figure 3B). This effect was modest yet statistically significant, leading to approximately 25-30% decrease in cell growth after 96 hours. This growth pattern was also observed using a micro-electronic biosensor system (designated 'real-time-cell-analyser', RTCA [21]) that allows real-time monitoring of cell growth in-vitro (Figure 3C). Cellular migration was monitored using an in-vitro transwell system. Mir-376a and mir-376c transfected cells showed significantly attenuated migration through a transwell membrane 24 hours after seeding relative to pTER-transfected control cells (Figure 3D). Migration was also monitored using the real-time-cell analyzer, this time assessing cell density following passage through a membrane as described in [22]. Whereas pTER-transfected control melanoma cells exhibited a time-dependent migration through the membrane, the mir-376a and mir-376c transfected cells showed almost no migration through the membrane within a 24 h period (Figure 3E).

**Figure 4:**
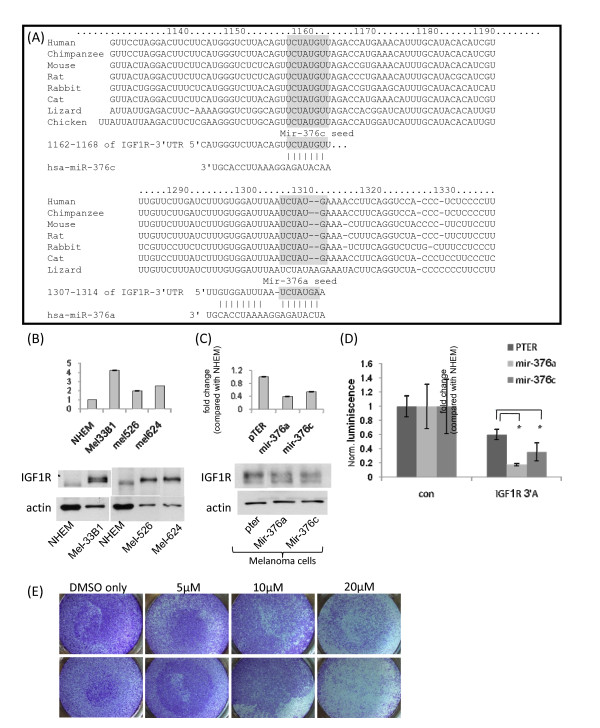
** Establishment of IGF1R as a target of mir-376a/c.(A)** The 3'UTR of the IGF1R gene showing potential binding sites for mir-376a/c (marked in grey). The sequence conservation from human to lizard in the vicinity of mir-376c and 376a seed sequences and the putative interaction of the miRNAs and the mRNA are shown (taken from TargetScan at http://www.targetscan.org/). **(B)** IGF1R mRNA and protein levels in NHEM and several melanoma cell lines using qRT-PCR and Western blot, respectively. **(C)** IGF1R mRNA and protein levels in pTER-transfected and mir-376a/c-transfected mel33B1 melanoma cells using qRT-PCR and Western blot, respectively. **(D)** Melanoma cell lines stably transfected with pTER, mir-376a or mir-376c were transiently transfected with a control-luciferase vector or with a vector containing luciferase attached to an element of the 3'UTR of the IGF1R gene containing the putative binding sited for mir-376a/c (designated IGF1R 3'A). The ratio of expression of luciferase/renilla was normalized relative to control-transfected cells for each of the cell lines. * signifies p < 0.05 using two-way ANOVA. **(E)** The migration of control pTER-transfected melanoma cells was assessed in the absence and in the presence of varying doses of AG-1024 using the in-vitro transwell method. Representative micrographs of the transwell membrane are seen. This experiment was repeated three times.

Bioinformatic analysis using several web-based tools showed that miRNA-376a and miRNA-376c have putative binding sites at the 3'UTR of IGF1R (Figure 4A), a tyrosine kinase receptor long known to be implicated in melanoma tumorigenesis and progression [23]. The putative binding site of mir-376c is classified as ‘7mer-8mer binding’, and that of mir-376a is classified as ‘8mer binding’. Both putative binding sites are located within the first fifth of the IGF1R 3'UTR. Theoretically, mir-376a could generate a stronger interaction with the IGF1R 3'UTR through additional nucleotide pairing beyond the "seed" sequence [24,25] (Figure4A).

As expected, both mRNA and protein levels of IGF1R were higher in melanoma cell lines than in normal melanocytes (Figure 4B). Stable expression of mir-376a or mir-376c led to a decrease in IGF1R levels both at the mRNA and at the protein levels (Figure 4C). In order to determine whether IGF1R is a direct target of miR-376a/c, we used a commercial plasmid containing the first ~2800 nucleotides of the IGF1R 3'UTR cloned downstream to the luciferase reporter gene. This vector was then introduced into melanoma cells over-expressing mir-376a, mir-376c or a control vector (designated pTER). Introduction of the IGF1R-3'UTR-luciferase vector into pTER-transfected control cells led to a ~40% decrease in the level of luciferase expression relative to the same cells following introduction with a control luciferase vector. This probably reflects the negative regulatory action of endogenous miRNAs within the melanoma cells on this 3'UTR. Introduction of the IGF1R-3'UTR-luciferase vector into mir-376a-transfected or mir-376c-transfected cells led to a significant ~83% and ~65% decrease in the level of luciferase expression relative to the same cells following introduction with a control luciferase vector, respectively, indicating that the stable expression of both miRNAs leads to further significant down-regulation on the 3'UTR of IGF1R (Figure 4D), thus establishing IGF1R as a target of both mir-376a and mir-376c.

To assess whether the down-regulation of IGF1R by mir-376a and mir-376c could account for the observed biological phenotype in these cells, IGF1R was pharmacologically inhibited using the commercially-available IGF1R inhibitor AG-1024 [26]. IGF1R inhibition by AG-1024 pheno-copied the decrease in migration seen following over-expression of either mir-376a or mir-376c using the same experimental system, in a dose-dependent manner (Figure 4E). The administration of AG-1024 to melanoma cells over-expressing either mir-376a or mir-376c did not lead to a further decrease in their migration (results not shown), suggesting that the IGF1R axis could not be further modulated to decrease migration. AG-1024 did not lead to decreased cellular proliferation in either the control cells or the cells over-expressing mir-376a or mir-376c, suggesting that the modest effect of these miRNAs on cellular growth is mediated through different mechanisms.

## Discussion

We show here that miRNAs from a large cluster on chromosome 14q32 are significantly down-regulated or absent in melanoma cell lines, benign nevi and melanoma samples relative to normal melanocytes. This may suggest that their expression is lost along the transformation process of normal melanocytes into malignant cells. This resembles the well-known observation that the mutated form of B-RAF, which characterizes 40-60% of melanoma patients [27], can already be detected in benign pigmented nevi as well [28]. It is well-known that an acquired mutation in B-RAF is not sufficient for tumorigenesis [29]. We hypothesize that in a similar manner, the loss of expression of this miRNA cluster occurs already in the benign phase, but contributes to tumorigenesis and metastasis only upon the acquisition of additional genetic and cellular abnormalities.

The miRNA cluster on chromosome 14q32 has been shown to be down-regulated in ovarian cancer ([7], gastro-intestinal stromal tumors (GIST) [8] and gliomas [9], and aberrations in chromosome 14 have been implicated in many types of cancer (reviewed in [9]). In fact, this region was already dubbed 'the largest miRNA tumor suppressor cluster [9]. A recent review summarized the growing body of literature connecting this region to cancer in many sites [30], yet until now, it has not been implicated in melanoma.

Several analyses of miRNA arrays in melanoma have recently been published [4-6], all in agreement that only several miRNAs are differentially expressed between normal melanocytes and melanoma cell lines or samples. Neither work pointed to the almost complete disappearance of miRNA expression from this cluster. This is most likely due to methodological differences between the different works. Some of the chromosome-14q32-miRNAs were expressed in very low amounts in normal melanocytes, thus perhaps ‘evading’ detection with miRNA arrays of lower sensitivity than the one used in our current work, whereas at least ten miRNAs from the cluster were expressed in higher levels than the median expression level in the array. It is important to emphasize that the expression pattern of chromosome-14q32-miRNAs and maternal transcripts were consistently seen in all normal melanocyte samples examined by us from several different batches, using both the micro-array technique and qRT-PCR. Indeed, Stark et al. characterized the ‘melanoma miRNAome’ by performing deep sequencing of cell lines derived from normal melanocytes, melanoblasts, melanoma and a large congenital nevus, and also demonstrated that Chromosome-14q32-miRNAs are expressed in normal melanocytes but not in any melanoma cell lines [31], in complete agreement with our current work. Moreover, Philippidou et al. also observed that both mir-127-3p and mir-376c are down-regulated in a metastatic cell line relative to their expression in the primary tumor from the same patient [5], again in agreement with our current observations.

Genetic analysis in mice elegantly showed that a maternal deletion of the IG-DMR region could lead to a shut-down of the expression of genes from the maternal chromosome, thus rendering the expression pattern from this chromosome to be 'paternal-like' [11]. Our copy number assay indicates that LOH of the IG-DMR or complete absence of two copies of this region occurs in less than half of the cell lines examined. Our results are in line with published results, showing that 20% of the melanoma cell lines exhibit copy number losses in miRNA genes in chromosome 14q32 [20]. Nonetheless, LOH of the IG-DMR region is clearly not the sole mechanism underlying this miRNA cluster shut-down. Interestingly, an LOH spanning approximately 1.1 Mb in the same region on chromosome 14q32 was found to characterize many cases of neuroblastoma [32], a neoplasm derived from neural crest cells, the precursor cells from which mature melanocytes develop as well.

Recent studies suggested that the expression of Dlk1-Dio3 transcribed miRNAs is essential for maintaining full pluripotency of induced pluripotent stem cells (iPSCs), and that this expression is in fact the most significant discriminator between fully pluripotent and partially pluripotent inducible cells [17,33]. These works, albeit descriptive in nature, again point to the cardinal role of this large miRNA locus on the fine interplay between differentiation, pluripotency and transformation.

We observed that only a combination of de-methylating agents and HDAC inhibitors (designated 'epigenetic modifiers' in short) could lead to re-expression of two maternally expressed genes and only very few miRNAs from this cluster. We could not find a correlation between the number of copies of the IG-DMR region and the potential or the extent of re-expression following treatment, suggesting that epigenetically ‘switching on’ a silenced allele is feasible whether there are two alleles in the cell or only one.

The observed increase in the levels of acetylated-histone-3 DNA in three different loci within the regulatory regions following treatment with epigenetic modifiers suggests that epigenetic alterations takes part in silencing of this cluster. The observation that only a few miRNAs from the cluster could be re-expressed after treatment with epigenetic modifiers was somewhat surprising. Four of these miRNAs (namely mir-127-3p, mir-136, mir-376a and mir-376c) were shown to be down-regulated but not completely silenced in nevi and melanomas. These results, taken together, suggest that the regulation of the expression of miRNAs from this cluster is complex and multi-leveled. Whereas previous results suggest that the IG-DMR is an important regulatory 'switch' in this region [34], our work suggest that it is by no means the only one. One can postulate that specific miRNAs within this large cluster have their own individual 'switches', and indeed such a switch has been suggested for mir-127 [10], also shown to be up regulated in our work in response to epigenetic modifiers.

Ectopic expression of mir-376a and mir-376c had a modest yet significant effect on cell growth, but a profound effect on cellular migration in-vitro. Indeed, it has already been suggested that melanoma proliferation and migration are controlled through different regulatory circuits [35]. The Insulin growth factor 1 receptor was recently shown to be constitutively activated in melanoma cells in an autocrine fashion [36]. Insulin-like growth factor 1 (IGF-1) was shown by others to significantly increase melanoma cell migration in-vitro through activation of the IGF1R. IGF1-stimulated migration required PI3K activation but was independent of MAPK/ERK signaling [37]. In our experimental system, IGF1R levels were higher in melanoma cell lines than in normal melanocytes, and the ectopic expression of mir-376a and mir-376c led to down-regulation of the receptor. Luciferase reporter assays indicate that, as bioinformatically predicted, mir-376a and mir-376c directly target IGF1R. Pharmacological inhibition of IGF1R pheno-copied the decrease in migration seen following mir-376a and mir-376c over-expression, suggesting that down-modulation of the IGF1R signaling pathway may be responsible for the observed anti-migratory effect of these miRNAs in melanoma cell lines.

Other miRNAs have been shown to down-regulate IGF1R. For example, mir-145, a known tumor-suppressor-miRNA, was shown to inhibit the IGF1R axis by targeting both IRS-1 and IGF1R [38]. Recently, mir-493 (curiously also located on chromosome 14q32) was shown to be capable of inhibiting liver metastasis in a colon cancer model by targeting IGF1R [39]. Nonetheless, the inhibition of IGF1R by mir-376a and mir-376 has not been described before.

## Conclusions

We show here that a large miRNA cluster on chromosome 14q32 is silenced in malignant melanoma. This cluster has been implicated in many cancers, as well as in differentiation and in determination of pluripotency, but not in melanoma so far. This silencing may involve genetic or epigenetic mechanisms, and can partly be reverted in-vitro using epigenetic modifiers such as de-methylating agents and HDAC inhibitors. Re-expression of two miRNAs from this cluster, namely mir-376a and 376-c, attenuate melanoma proliferation and migration. Both these miRNAs target IGF1R.

IGF1R has already been implicated in melanoma almost 20 years ago [40], and data concerning its exact role in the pathogenesis of this disease is rapidly accumulating (reviewed in [23]). Eight years ago the IGF1/IGF1R pair was shown to lead to melanoma migration [37], and in fact IGF1R was recently identified as a potential target in melanoma using a phosphoproteomic screen [41]. Last, in-vitro work showed that resistance to B-RAF inhibition could be overcome by simultaneously co-targeting MEK and IGF1R/PI3K, and that indeed IGF1R levels are increased in human tumor sample following the acquisition of resistance to B-RAF inhibition, consistent with a role for IGF1R/PI3K-dependent survival in the development of such resistance [42]. More specifically, the possibility of targeting the IGF1R by siRNAs in B-RAF-mutated melanoma cells was also already suggested several years ago [43].

The work presented here demonstrates that mir-376a and mir-376c negatively regulate IGF1R, and suggests that aberrations in this regulatory mechanism, in the form of down-regulation of mir-376a/c, take part in melanoma progression and metastasis. In lieu of growing interest in this pathway in relation to B-RAF inhibition, our work may, in the future, contribute to further understanding of the phenomenon of resistance to B-RAF inhibition.

## Methods

### Cells cultures and reagents

Melanoma cell lines were generated directly from metastatic melanoma lesions of patients at the Surgical branch of the NIH (mel526, mel624, mel938, mel33A1, mel33B1;[44,45]) or at the ‘Ella institute for melanoma research’ at the Sheba Medical center (eng-003, 14PA, 15AY [46]). The cell lines were grown in DMEM medium supplemented with 10% fetal bovine serum (FBS), 1% Penicillin–Streptomycin antibiotics, 1% L-glutamine and 2.5% HEPES solution (Biological Industries, Kibbutz Beit Haemek, Israel). Normal human epidermal melanocytes (NHEM) were purchased (three different batches throughout the period of research) from Promocell (C-12400; Promocell, Germany) and grown in melanocyte growth medium (C-24010; Promocell) according to manufacturer's instructions. NHEM were maintained in culture for up to 5 cycles.

AG-1024 (a commercially-available IGF1R inhibitor) was purchased from Calbiochem- EMD Biosciences (La Jolla, CA, USA).

### Cloning

Both mir-376a and mir-376c pre-miRNAs were cloned into the pTER plasmid [47,48]. It is to note that there are two miRNA genes, mir-376a-1 and mir-376a-2, coding identical mature miRNAs, that are indistinguishable. Briefly, both sense and anti-sense oligos of the pre-miRNA were synthetically synthesized (Sigma, Israel). Sequences were taken from the miRBase data base as follows:

Mir-376a sense primer:

5'TAAAAGGUAGATTCTCCTTCTATGAGTACATTATTTATGATTAATCATAGAGGAAAATCCACGTTTTC-3'

Mir-376c sense primer:

5'-AAGGTGGATATTCCTTCTATGTATGTATTTATGGTTAAACATAGAGGAAATTCCACGTTTT-3'

GATC (the complementary sequence to BglII digested) was added to the 5' end of the sense oligo, and TCGA (the complementary sequence to HindIII digested) was added to the anti-sense oligo. Sense and anti sense oligos were Annealed and ligated into the pTER vector digested with BglII and HindIII.

The IGF1R-3'UTR Lucifrase reporter plasmid (clone A HmiT009523-MT01) and control plasmid (no 3'UTR; CmiT000001-MT01) were purchased from GeneCopoeia, Inc. (Rockville, MD).

### Generation of stable melanoma cell lines

Cells were transfected with purified DNA plasmids with the Lipofectamine™ 2000 Transfection Reagent (Invitrogen, Carlsbad, CA), according to the manufacturer protocol. 24 hours after transfection, Zeocin antibiotic (300μg/ml) was added to the cells for selection. Following selection, the stable ectopic expression of mir-376a/c was repeatedly assessed using qRT-PCR.

### Tumor samples

Formalin-fixed-parrafin-embedded (FFPE) samples of benign nevi or primary cutanous melanoma were obtained from the pathology institute at the Sheba Medical Center. The initial diagnosis of melanoma and the histological type was verified by a pathologist on the hematoxylin-eosin–stained slides, performed on the first and/or last sections of the sample. The tumor or nevus was macro-dissected from the slide in the cases in which the sample contained normal tissues as well, based on demarcations delineated by the pathologist. The study was approved by the ethics committee of Sheba Medical Center and conducted in adherence to the Declaration of Helsinki protocols.

### RNA extraction

Total RNA was extracted from cell lines using Ambion *mirVana*™ miRNA Isolation Kit (Ambion, Austin, TX). Total RNA from 10 sections of 5 μm FFPE tissues was extracted using the Qiagen miRNeasy FFPE kit (Qiagen, Germantown, MD). Quantity and quality were evaluated using a Nanodrop ND-2000 (Thermo Scientific, Waltham, MA) with inclusion criteria of A260/A280 ≥1.8. For positive control, a commercial sample of placental miRNAs was used (Ambion, Austin, TX).

### miRNA micro-array experimentation and analyses

miRNA expression profiling was performed using Agilent’ Human miRNA Micro-array system V2 and later V3 (Agilent Technologies, Santa Clara, CA) with probe sets for approximately 850 human miRNAs (taken from the ‘miRBase’ database) according to the manufacturer’s protocol . In brief, 100 ng of total RNA were fluorescently-labeled with Cyanine 3-pCp, and hybridized onto the arrays for 18–20 h at 55 °C. Slides were scanned in an Agilent micro-array scanner G2565BA and the images obtained were processed with Feature Extraction Software 9.5.3.1 (Agilent, Santa Clara, CA). Cluster analysis was done on the normalized, log transformed values with the k-means algorithm using the MATLAB software (MathWorks, Natick, MA).

### Quantitative real time PCR

#### MiRNA

Quantification of miRNAs by *TaqMan* MicroRNA assays (Applied Biosystems, Carlsbad, CA) was carried out using 10 ng of RNA. Target miRNA expression was normalized between samples based on the expression levels of Rnu19 or Rnu48. The ΔΔCT method was used to calculate the expression values.

#### mRNA

IGF1R mRNA levels was assessed with the TaqMan® Gene Expression Assay (ID Hs99999020_m1 IGF1R). Gene expression was normalized between different samples based on the values of Rplpo expression.

#### Copy number assay

Total cellular DNA was extracted using genomic DNA extraction kit (iNtRON biotechnology, Seongnam, Korea). Quantification of DNA by *TaqMan* Copy Number assays was carried out using 10 ng of DNA with the primers Hs03889256_cn, Hs03874180_cn, Hs03877160_cn (Applied Biosystems, Carlsbad, CA). Genomic Rnase P region served as a reference assay. Analyzes were done using the 'CopyCaller^TM^ software' (Applied Biosystems, Carlsbad, CA).

### Determination of mRNA levels by RT-PCR

Reverse transcription-polymerase chain reaction (RT-PCR) was performed using the Verso thermo-scientific kit (Thermo-Scientific, Waltham, MA). PCR primers are listed (Table 4).

**Table 4:** Primer sequences used for assessment of mRNA levels by qRT-PCR

**Gene name**	**Forward**	**Reverse**
β-actin	CCTGGCACCCAGCACAAT	GCCGATCCACACGGAGTACT
Meg3	TGCGGAAGAGGCCCTGAT	GTCCAGAGTCTCTGGGTCCA
Meg8	CAGTGTTGCCTGGGTCTGA	ATCCCCTTGAAAGAGCAGGA
Rtl1	CCTGCCCTGCGTCAGAACCG	TGCTGGAGACAGGGAGGCGT
Dio3	TGGTGGTCGGAGAGGGCGAG	CGGTTGTCGTCGGACACGCA

### Treatment with epigenetic modifiers

Cells were seeded at 50% confluence 8 hr prior to treatment with 5-Aza-2'-deoxycytidine (5-Aza 10 μM; Sigma-Aldrich, Rehovot, Israel) and valproic acid (VPA 1.5 mM; Sigma-Aldrich, Rehovot, Israel) or phenylbutyric acid (PBA 3 mM; Sigma-Aldrich, Rehovot, Israel). The drugs were continuously administered by replacing the medium every 24 h for 5 days.

### Chromatin-immunoprecipitation (ChIP) assay

14PA melanoma were cells treated with 5-Aza and PBA and subjected to a ChIP assay using a modified protocol as decribed in [49]. In short, cells were incubated with formaldehyde, washed, centrifuged and resuspended in 1% SDS-containing buffer and then sonicated. Extracts were immunoprecipitated with anti-acetylated histone H3 antibody (Millipore, Billerica, MA) overnight at 4°C. Quantitative analysis was performed by real-time PCR with *TaqMan* primers as follows: Hs03889256_cn and Hs03874180_cn, both located within the 8 kb-long IG-DMR region (approximately 15 kb upstream of Gtl2 and 70 kb downstream of the Dlk1 promotor); and Hs03877160_cn, located approximately 700 bp upstream of mir-127 (Applied Biosystems, Carlsbad, CA).

### Luciferase assay

Luciferase assay was performed 48 h post transfection with a control vector or a vector containing part of the 3'UTR of the IGF1R using the Dual Luminescence Assay Kit (GeneCopoeia, Inc., Rockville, MD) as described by the manufacturer.

### Determination of protein expression level by western blotting (WB)

WB was performed using monoclonal primary specific antibodies (IGF1Rβ c20 CS-713, Santa Cruz Biotechnology, USA, and Beta Actin AC-15, Abcam, UK) as perviously described [50].

### Cell growth and migration in-vitro

#### Crystal violet

Melanoma cells (5 × 10^3^) were seeded in a 96-well plates and viable cell counts were monitored from seeding time to 96 h. The cells were fixated with ethanol 70% and stained with crystal violet 0.1%. The color was extracted using 1% triton x-100 and absorption was read at 550 nm. Each experiment was performed in quadruplicate, and repeated at least three times.

#### Transwell migration

Melanoma cells (2 × 10^5^) were seeded in the upper wells of a Transwell migration system on ThinCerts^TM^ inserts with 8-μm membranes (Greiner-bio-one, Frickenhausen,Germany) in DMEM supplemented with 0.1% FBS. The lower well contained the same medium with 10% FBS. After 24 hours of incubation, the upper well content, which contained non-migrating cells, was vigorously removed using cotton swabs. The cells that migrated through the membranes were fixated with 70% cold Ethanol, stained with crystal violet 0.1% and photographed using the light microscope. Each experiment was performed in triplicate, and repeated three times.

#### Real-time-cell-analyser (RTCA®)

Melanoma cells were seeded in the xCELLigence^TM^ DP system (Roche Diagnostics GmbH, Mannhein, Germany) and incubated for 1–5 days. For monitoring growth, data were collected every 20 min automatically by the analyzer as described in [21]. For verification, a cellular growth curve was also obtained using the crystal violet technique described above. For monitoring migration, cells were seeded in the upper chamber in the normal culture medium of the respective cell line with 0.1% FBS. This upper chamber was then placed on the lower part of the CIM-device containing growth medium supplemented with 10% FBS as an attractant. Migration of the cells was followed for 24 h by tracking changes of the impedance signal in a CIM-plate measured on the opposing side of the membrane as described in [22]. Each experiment was performed in duplicates and repeated twice.

### Statistical analysis

Statistical significance was determined using the Student’s *t*-test or using two-way ANOVA. For a single comparison, a p-value < 0.05 was considered significant. For multiple comparisons, a p-value < 0.0032 was used, taking into account multiple comparisons using the method of false detection rate (FDR).

## Abbreviations

ChIP, Chromatin immunoprecipitation; DMR, Differentially methylated region; HDAC, Histone de-acetylase; IG-DMR, Intergenic differentially methylated region; IGF1R, Insulin growth factor 1 receptor; LOH, Loss of heterozygosity; miRNA, micro-RNA; NHEM, Normal human epidermal melanocytes; RTCA, Real-time cell analyzer.

## Competing interests

There are no financial or non-financial competing interests to declare.

## Author contributions

LZ performed the molecular biology and tissue culture experiments; RA performed the micro-array experiments (with LZ); AB and DB performed the experiments with the pathological material; RN performed the bioinformatic and statistical analyses; LZ, YS, DA and RLA designed the experiments, analyzed the data and prepared the manuscript. All authors read and approved the final manuscript.

## Supplementary Material

Additional file 1 The expression levels of miRNAs in normal melanocytes and melanoma cell lines. Click here for file

Additional file 2 The expression levels of miRNAs in normal melanocytes, benign nevi and melanoma samples. Click here for file

Additional file 3 The expression levels of miRNAs in melanoma cell lines in response to epigenetic modifiers. Click here for file

## Ackowledgements

We would like to thank Dr. Tami Rubinek for her generous gift of AG-1024 and Dr. Guy Amit for his help in the bioinformatical analysis of the micro-array results. This work was supported by grants from the Israeli Cancer Association (ICA), The ‘Morasha’ program of the Israel Science Foundation (grant No. 1499/09) and the Chief Scientist Office at the Israeli Ministry of Health.

Dr. Leibowitz-Amit is a recipient of a Dr. Edward H. Kass fellowship from the American Physicians Fellowship for Medicine in Israel, a recipient of the ‘Stewart Rahr – MRA Young Investigator Award’, and a member of the 'Talpiot Medical Leadership program' at the 'Sheba Medical Center'.
